# Freshwater fisheries conservation can increase biodiversity

**DOI:** 10.1371/journal.pone.0233775

**Published:** 2020-05-27

**Authors:** Declan Butorac, Paulo Santos, Phousavanh Phouvin, Francois Guegan

**Affiliations:** 1 Environmental Management, Monash University, Clayton, Vic, Australia; 2 Dept Economics, Monash University, Caulfield, Vic, Australia; 3 Dept of Fisheries, National University of Laos, Vientiane, Lao PDR; 4 WWF - Laos, Vientiane, Lao PDR; Universidade Federal de Mato Grosso do Sul, BRAZIL

## Abstract

We evaluate the impact of a fisheries management program centered on the definition of Fish Conservation Zones on biodiversity, measured as the number of species caught in the last 12 months. Data comes from a set of 32 villages in central Lao PDR, of which half participated in the program, and the remaining are a set of matched control villages. The estimated causal effects are large (an increase between 29 and 32 species) and robust to the potential importance of unmeasured confounders. We also show that initial conditions may matter, as the program seems particularly effective in villages with high probability of participating in the program. These results are particularly important given the paucity of evidence regarding the impact of conservation programs on biodiversity, particularly in the context of freshwater ecosystems. Further directions of research suggested by these results are discussed.

## Introduction

There is a widely accepted recognition that freshwater ecosystems are extraordinarily rich in terms of biodiversity: representing approximately 2.3% of the global land surface, fresh waters host approximately 9.5% of the described animal species [1]. It is also widely recognized that such richness is under disproportionately increased pressure, from both old and new threats [1, 2]. As a result the rate of decline in biodiversity in freshwater systems has outpaced that of terrestrial ecosystems [3, 4].

Adding to this diagnostic, there is also a suspicion that approaches to conservation adopted in terrestrial ecosystems, based on the definition of high habitat quality that can be well defined and bounded (“fortress conservation” in the words of [1]), are difficult to transfer to conservation of freshwater systems: protection of a particular habitat may require control of upstream drainage areas and, if migratory behavior is important, also of the areas downstream. The whole catchment becomes the natural boundary, but conservation encompassing this scale is rare [5]. Perhaps reflecting these difficulties, there is only a small number of examples of freshwater conservation initiatives relying on no-take areas: see, for example, [6] for Cambodia, [7] for the countries around Lake Victoria in east Africa, [8] for India, [9] for The Philippines and, finally, [10, 11] for Lao People’s Democratic Republic (hereafter, Lao PDR), perhaps the oldest documented example, and also the one where our analysis is based.

Although these examples suggest that the difficulties in implementing freshwater conservation are not universal or unsurmountable, they tell us little about whether they “work” in the sense that they contribute to avoid reductions in biodiversity. This is a shortcoming that is shared with earlier conservation work: in the mid-2000s, [12] asked whether biodiversity conservation investments were “money for nothing?”, and [13] suggested we were “shooting in the dark”. Almost 10 years later, similar questions are still being asked: [14] asks why is the evidence on win-win interventions so poor while [15] decry the lack of evidence on the impacts of biodiversity conservation on poverty (and vice-versa) as the missing Randomized Control Trials. The solution advocated by these authors is to mainstream impact evaluation into the design and implementation of conservation programs [16, 17], with the objective of addressing the identified knowledge gaps in terms both of conservation effectiveness.

The work presented in this article contributes to that growing literature. Our data comes from Lao PDR, a landlocked country where the Mekong, one of the richest and most threatened river basins in terms of freshwater biodiversity [1, 18], seems to never be far away. Building on indigenous knowledge, that guided their initial definition and enforcement, fish conservation zones or FCZs (as these no-take areas are locally known) have been incorporated in the practice of multiple programs, even while their impacts remained largely unknown. Here, we present estimates of the causal impact of the establishment of FCZs on biodiversity, by evaluating the impact of a fisheries management program that is centered on their definition.

## Methods

### Freshwater fisheries conservation in Lao PDR: ComFishIII

There is a long history of local knowledge about the definition of no-take areas as a mechanism to manage local fisheries in Lao PDR [11]. Those areas rely on the exclusion of fishing in deep pools—parts of the stream where water level is high enough during the dry season so as to provide a suitable habitat year round, and where the slower flow creates a suitable place for spawning [19–21].

This knowledge has been incorporated in different fisheries conservation programs in Lao PDR, including the third phase of the Community Fisheries program (ComFishIII), implemented by WWF Laos, during the period 2012-2015, in collaboration with the Ministry of Agriculture and Forestry (MAF) and with local participation of village authorities. Its guidelines [22] reflected both the recognition of the importance of local knowledge regarding fisheries conservation [11] and a legal framework that emphasizes the co-management of local fisheries [23].

Its implementation involved a series of decisions by WWF and MAF that, typically, started with the preparation of a list of potentially eligible villages, presented to WWF by the District representatives of MAF. This list reflects perceptions of the importance of fishing (number of households who fish, existence and importance of alternative livelihoods) and implicitly assumes that these criteria reflect a healthy ecosystem, as no baseline data on biodiversity is ever collected. This initial submission is then followed by a series of steps that end with the development of local regulations to manage fisheries (see Fig A in S1 Appendix).

The first of these steps, consensus building, is the one that matters most to understand program placement. It includes an analysis of the potential importance of fishing to local livelihoods, the social context of local fisheries (including analysis of conflicts around local fisheries and of social cohesion, proxied by village history, including recent episodes of resettlement, and ethnic heterogeneity) and, finally, a preliminary discussion regarding willingness in changing the way that local fisheries are managed. It also includes the clarification of whether deep pools exist (as the creation of FCZ hinges on its existence). This step typically involves one or more visits by WWF staff (facilitated by local government authorities), from which then emerges a final list of eligible villages.

The next steps involve the drafting and re-drafting of local regulations. The approval of these regulations at local level (including informing neighbouring villages and government bodies, such as police, of their existence) precedes the formal approval by the District Governor. The process ends with the advertising of the new fishing regulations as well as the limits of the newly created FCZ (usually, through the use of public signboards).

### Statistical analysis

We follow Rubin’s potential outcome model of causal inference [24] and start by stating that each unit of analysis in the population of interest (village) has two potential biodiversity outcomes (number of species), one when ComFishIII is implemented (*Y*_*i*_(1)) and another one in its absence (*Y*_*i*_(0)). The actually observed outcome (*Y*_*obs*_) can be expressed as a combination of these two potential outcomes according to the expression:

$$Y_{obs}=T_{i}Y_{i}\left(1\right)+\left(1-T_{i}\right)Y_{i}\left(0\right)$$ (1)

where *T*_*i*_ = {0, 1} indicates treatment status (= 1 if participating in program, 0 otherwise), making clear that we cannot observe counterfactual potential outcomes—for example, [*Y*_*i*_(0)∣*T*_*i*_ = 1] defined as the outcome of a treated unit had it not been treated.

If nothing can be done at individual level, at population level we can quantify the Average Treatment on Treated (*ATT*) as

$$\begin{array}{cc} ATT & =E[Y_{i}\left(1\right)-Y_{i}\left(0\right)|T_{i}=1]\\ & =E\left[Y_{i}\left(1\right)|T_{i}=1\right]-E\left[Y_{i}\left(0\right)|T_{i}=1\right] \end{array}$$ (2)

where *E*[.] is the expectation operator and *T*_*i*_ = 1 indicates that the unit participated in the program. If a program is randomly placed then, by design, units participating in the program will be (on average) identical to those that do not participate, hence

$$E\left[Y_{i}\left(0\right)|T_{i}=1\right]=E\left[Y_{i}\left(0\right)|T_{i}=0\right]$$ (3)

and the *ATT* takes the simple form of *E*[*Y*_*i*_(1) ∣ *T*_*i*_ = 1] − *E*[*Y*_*i*_(0) ∣ *T*_*i*_ = 0]. When the program is purposefully placed and/or participation is voluntary, there is no *a priori* certainty that Eq 3 is satisfied, raising the need to consider quasi-experimental designs that adequately address this problem—see [25] for a review of different approaches in the context of environmental programs.

Matching, the approach used here, aims to eliminate the effect of any imbalance in the distribution of potential outcomes (and the effect of confounders on potential outcomes) on the estimates of *ATT*. In these designs, the counterfactual is then expressed as

$$E\left[Y_{i}\left(1\right)|T_{i}=1,X\right]=E\left[Y_{i}\left(0\right)|T_{i}=0,X\right]$$ (4)

which formalizes the Conditional Independence Assumption (CIA), ie, that once we account for the effect of all potential confounders *X*, program participation is *as if* random in the sense that it is independent of potential outcomes. [26] shows that the effect of *X* can be summarized by an appropriate specification of the probability of participating in the program, also known as the propensity score. Given that the allocation of many environmental programs is not usually randomized, matching on the propensity score is a widely supported alternative approach (see [27] for a discussion of the utility of this approach in this context). As usual in this literature, we base our analysis of the statistcial significance of our estimates on bootstrapped standard errors, as these allow us to account for the additional noise introduced by the initial statistical modelling of the participation decision [28].

The CIA is a strong assumption as it essentially requires us to accept that no relevant confounder was left out of the estimation of the propensity score, the function that we use to judge the similarity between treated and control units. Rather than claiming that such assumption is held, we follow the *ex ante* literature and present a form of sensitivity analysis that relies on the estimation of Rosenbaum bounds [29]. Intuitively, we answer the question “how important does the relative influence of unobserved confounders have to be to change our conclusion about the value of the program?”. More formally, we can express this idea by rewriting the probability of participation as

Prob(T=1∣X,u)=F(βX+γu) (5)

where *u* are the unobserved variables and *γ* is the effect of these variables on the participation decision; *F* is the CDF of *u*. If we assume that *F* = Λ, the odds-ratio that two individuals *i* and *j* will be treated is given by

$$\frac{\frac{P_{i}}{1-P_{i}}}{\frac{P_{j}}{1-P_{j}}}=\frac{P_{i}\left(1-P_{j}\right)}{P_{j}\left(1-P_{i}\right)}=\frac{exp(\beta X_{i}+\gamma u_{i})}{exp(\beta X_{j}+\gamma u_{j})}$$ (6)

But if matching is well done (ie, if CIA holds), this converts to *exp*(*γ*(*u*_*i*_ − *u*_*j*_)). Unconfoudedness means that either *u*_*i*_ = *u*_*j*_ (balance on unobservables, as under randomization) or *γ* = 0 (ie, *u* are not important in explaining participation, given we accounted for all relevant confounders *X*). If any of these is not true, then two observationally identical individuals will have different probabilities of getting treatment. Rosenbaum [29] shows that these probabilities (in terms of odds ratio) are bounded by:

$$\frac{1}{e^{\gamma }}\leq \frac{\frac{P_{i}}{1-P_{i}}}{\frac{P_{j}}{1-P_{j}}}\leq e^{\gamma }$$ (7)

where the interpretation of *e*^*γ*^ = 2 tells us that observationally similar units (in terms of *X*) could differ in terms of their probability of receiving treatment by as much as a factor of 2. The important question then is: even if true, would that change our estimates of the treatment effects?

Finally, we consider that differences in propensity score estimates between treated units (ie, differences in likelihood of participating in ComFishIII) can be interpreted as a measure of heterogeneity in initial conditions. We can use this intuition to examine heterogeneous treatment effects, using the approach suggested in [30], who define neighborhoods of treated units that are more homogeneous than the whole sample. This splitting of the original sample then allows for the analysis of the relation (trend) between the ATT estimate and the values of the propensity score, which is estimated using local polynomial regression. Differences in those local estimates would be suggestive of the importance of initial conditions on the magnitude of the treatment effect, with potential implications in terms of targeting, although care must also be taken in interpreting this link between initial conditions and outcomes as causal, given that in the context of programs that are designed locally, treated villages may exhibit other forms of heterogeneity (for example, with respect to the rules that are adopted).

### Constructing the control group

ComFish was not allocated randomly. We construct the control group by estimating the probability of participating in ComFish, given what we know about how the program was allocated, as described above. In the absence of a baseline survey, we use the Agricultural Census 2011/12 as the source of information for the estimation of the propensity score and the construction of the control group. Importantly, the Census contains information about a wide variety of village characteristics that might have conditioned the decision to promote ComFishIII, including the importance of fishing for local livelihoods. The process of constructing the control group followed four steps.

*Step 1: Identification of participating villages*. We focused on four provinces in Central and Southern Laos (Bolikhamxay, Khammouanne, Savannakhet and Champasak). Because we relied on the Agricultural Census 2011/12 as a source of pre-intervention information, we excluded those villages where implementation of the program started in 2011/12. In addition, several ComFishIII villages had to be dropped from the analysis, as they could not be identified in the Agricultural Census.

*Step 2: Identification of potential control villages*. The identification of a set of villages that are statistically identical to those that were included in the ComFishIII was based on the estimation of the propensity score. To satisfy the assumptions underlying this approach (see [26, 28]), in particular the CIA (Eq 4), we relied on the information collected through the Agricultural Census 2011/12. We had access to information on: 1) Importance of fishing and other livelihood activities; 2) Access to roads (and, with them, outside markets) and public services; 3) Importance of ethnic heterogeneity, as measured by the number of ethnic groups with more than 5% and 10% of the village population, as well as the fraction of the population that belongs to the first and second most important ethnic groups in the village; 4) Village history, notably their experience with resettlement; and 5) Location of administrative headquarters (District and/or Province). In the absence of detailed geographical information regarding access to freshwater, we limited the estimation of the propensity score to those villages that had a percentage of households who fish that was greater or equal to the minimum of this variable in ComFishIII villages (25%), which we took as a proxy for this determinant of program placement.

The estimates of the probability of participating in ComFish (ie, the propensity score), presented in S1 Appendix of Table A1, were obtained using the command –pscore– for Stata, described in [31]. The common support option was imposed. Once balance on covariates was achieved, and based on these estimates, we chose as potential control for each ComFishIII village the three non-participating villages with the closest value of the propensity score (ie, the three nearest neighbors).

*Step 3: Local validation of the list of matches*. The control group built in the previous step would be valid under the hypothesis that the Agricultural Census contains all information that might impact on this decision. Although the Agricultural Census contains a rich variety of information, this seems unlikely: for example, the Census does not contain information about the nearby existence of deep pools, on which ComFishIII conservation activities are based. To overcome this limitation, the list of potential matches was presented and discussed with Provincial Agricultural and Forestry Offices (PAFO), with the objective of collecting additional information (existence of deep pool, presence of other fishery development projects) that might impact on its inclusion as a possible control village. As a result of these discussions, we dropped from the control group those villages that had no deep pool close by or where other fisheries development projects were present.

An important concern when evaluating conservation programs is the potential importance of spillover effects [32]. This is particularly important when their success hinges on the importance of those same spillovers, as is the case of local no-take areas. (see [33] for a related discussion on the impacts of Marine Protected Areas). We used the available information on the extent of spillovers from previous conservation projects [20] to exclude from the control group those areas that could be indirectly impacted by villages participating in ComFishIII, by imposing a minimum distance of 20km between treated and control villages. This distance is approximately the double of previous estimates of the extent of spillover effects in this region [20].

**Fig 1:**
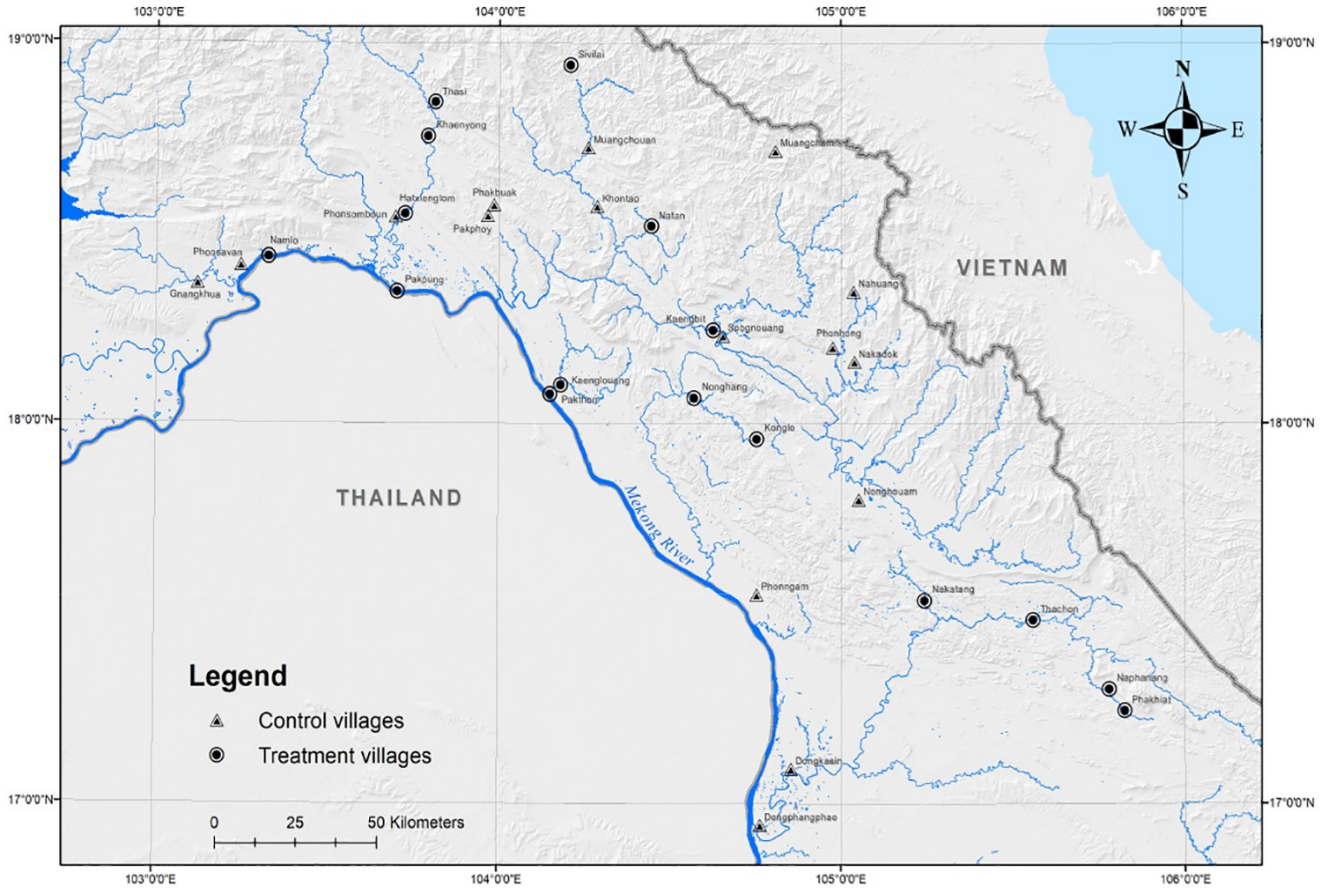
Location of treated (ComFishIII) and control villages.

*Step 4: Final list of control villages*. Based on the response and detail of the feedback from the Provincial Offices, a final list was constructed that is limited to two provinces (Bolikhamxay and Khammouane). We excluded Savannakhet because the response from PAFO was insufficient to proceed. We excluded Champasack because all treatment and control villages would be situated in the mainstream Mekong and, given the importance of migratory fish, we would not be able to minimise the importance of spillovers across villages. We also excluded those villages where the program was based on reservoirs. Finally, logistical difficulties (namely, impossible access at the time of the survey) limited the village list to 32 villages (from the original 36 identified in the previous steps), of which half are in ComFishIII (see Fig 1).

### Data on biodiversity

Local fishers (usually, the members of village fisheries committee) were presented with a list of 139 species likely to be found in the surveyed areas—see S1 Appendix—and asked to identify which species were caught in the previous 12 months. Interviews were conducted between February and March 2018, more than three years after the establishment of the last FCZ.

To facilitate identification, each species was described by a photograph of a typical specimen and its common Lao name. Interviewers, all junior staff from the Fisheries Department at the National University of Laos, were also instructed to provide additional assistance in case of doubt, by answering questions about the species (colour, typical size, etc). This data allows us to calculate species richness, a count of the number of species caught in the last 12 months, considered to be a good indicator of fisheries’ health [19]. This approach is similar to the one used in [34] to monitor a smaller number of threatened Mekong megafauna, also in Lao PDR.

There are several potential shortcomings to this approach, which are likely to be less important in the context of evaluating the impact of a conservation program. The first is that we do not measure species that are not caught or consumed, either because they are not part of the local diet or because they are protected. It is however unlikely that we would find large differences in food habits between treatment and control villages in this dimension given that we work in a relatively small area of the country.

The second is that differences in fishing practices (eg, restrictions on the use of nets with smaller mesh) may change the diversity of species caught. Existing studies that attempt to measure the effect of sampling technique on biodiversity seem to suggest greater diversity in fishing gear (ie, sampling techniques) leads to higher estimates of species richness (see [35, 36]). Although this is likely a topic that would benefit from more empirical studies, it is unlikely to be a major driver of any difference between treated and control villages: as shown in the next section (Table 1), ComFishIII is not associated with a much higher incidence of restrictions on fishing gear.

**Table 1:** Control vs ComFishIII villages.

Variable	Control		ComFishIII		Control—ComFishIII		
	mean	SD	mean	SD	mean	s.e.	p-value
A: village characteristics							
N households	103.1	55.79	112.5	62.89	-9.4	21.02	0.657
N households fishing	77.69	35.54	93.50	50.01	-15.81	15.33	0.312
N households fishing: main income	2.44	7.71	0.63	1.62	-1.81	1.97	0.372
N households farm	85.7	39.34	98.2	49.84	-12.5	15.87	0.438
N households with land	82.75	37.04	96.57	49.95	-13.81	7.75	0.382
N ethnic groups >5%	1.813	0.981	1.875	1.258	-0.062	0.40	0.877
N ethnic groups >10%	1.750	1.000	1.625	0.885	0.125	0.33	0.711
Share households main ethnicity	80.17	22.94	80.69	22.32	-0.52	8.00	0.950
Share households 2nd main ethnicity	10.94	12.46	12.48	14.25	-1.54	4.73	0.751
District/ Province HQ	0.00	0.00	0.06	0.25	-0.06	0.06	0.331
Resettled village	0.188	0.403	0.188	0.403	0.000	0.14	1.000
Village to be resettled	0.125	0.342	0.125	0.342	0.000	0.12	1.000
B: management rules							
FCZ	0.50	0.52	1.00	0.00	- 0.50	0.13	0.001
Restrictions - gear	0.88	0.34	1.00	0.00	-0.12	0.09	0.15
Restrictions - species	0.56	0.51	0.88	0.34	-0.32	0.15	0.05
Restrictions - quantity	0.25	0.45	0.44	0.51	-0.19	0.17	0.28
Monitoring	0.50	0.52	0.94	0.25	-0.44	0.14	0.005
Progressive penalties	0.75	0.45	1.00	0.00	-0.25	0.11	0.033

*p*-value for two sided *t*-test of difference in means (*n* = 32 villages, of which 16 participate in the Community Fisheries (ComFishIII) program.

One additional question is that recall data is potentially fraught with problems, given that memory is limited. However, the importance of fishing for the respondents (which is balanced across both types of villages) suggests that there is no reason to find a systematic difference in recall between treated and control units. Finally, the fact that the research team was not connected to the implementing agency (either WWF or the local government) and the fact that FCZ had been defined at least three years before the time of this evaluation should contribute to minimize any experimenter effects (positive or negative).

This approach also has important advantages, chiefly among them its low cost and the reduction of concerns about seasonality. Taken together, these advantages allow for the collection of data on a large number of treated and control villages, hence avoiding the shortcoming of case studies or very small samples.

## Results

### Treatment effects

**Fig 2:**
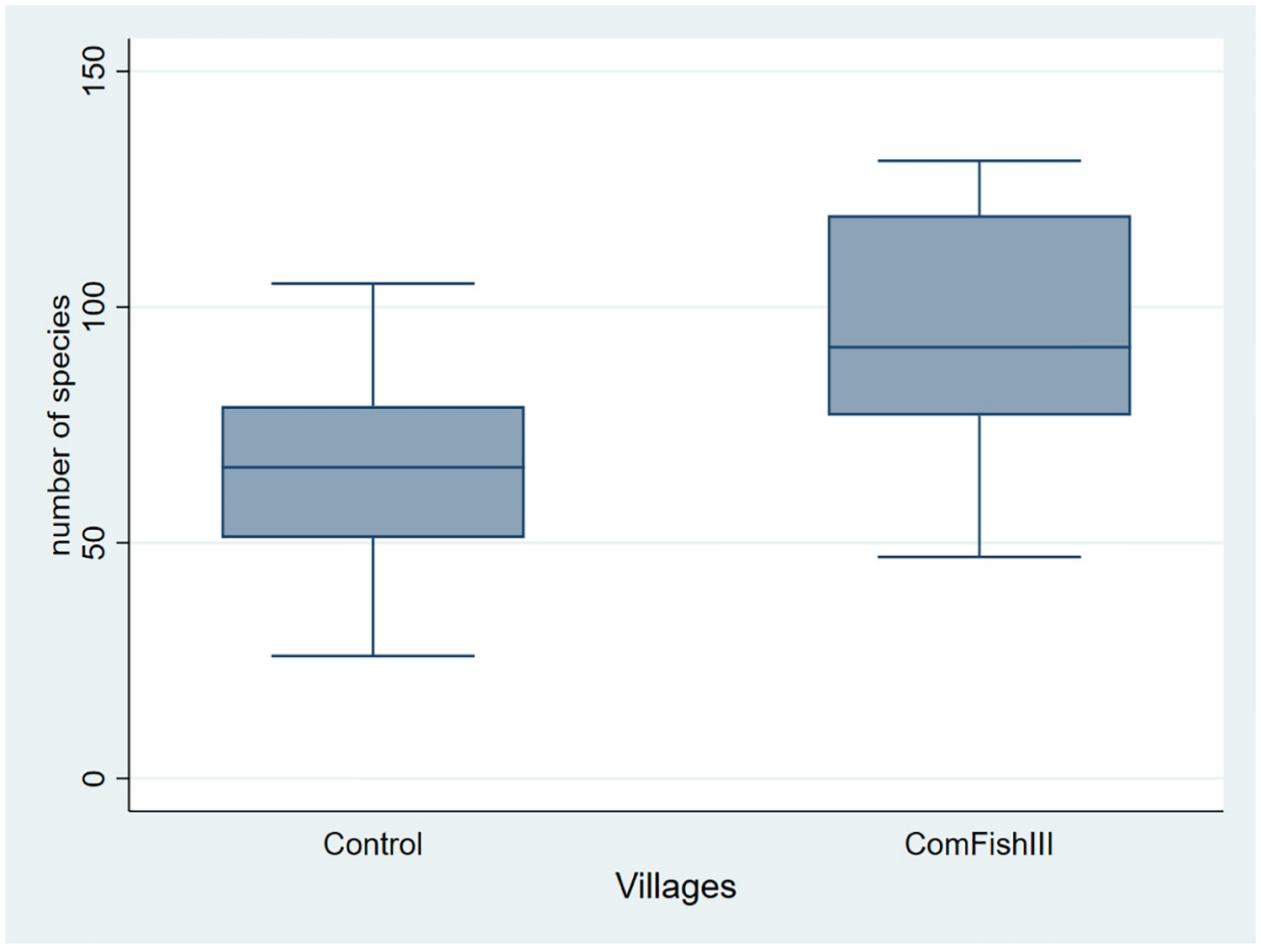
Number of species caught in treated (ComFishIII) and control villages Box plot with extreme values (minimum and maximum) and the three quartiles of distribution of number of species.

As shown in Table 1 we don’t find differences in (pre-program) village characteristics. In addition, the location of the 32 villages studied suggests a relatively random dispersion of the two types of villages across the territory (see Fig 1). We do observe, however, significant differences in (post-program) management rules: ComFishIII villages are much more likely to establish a local no-take area, to define restrictions on catching particular species, to pay people to monitor the enforcement of local regulations or to define progressive penalties for continued breaching of local regulations. Accompanying these differences, species richness is clearly much higher in program villages (Fig 2).

To rigorously quantify the average impact of the program, treated and control villages are first matched one-to-one on the propensity score using the nearest neighbor algorithm [31]. The results are presented in Table 2: the implementation of ComFishIII leads to a substantial increase in biodiversity, with the number of species caught in the last 12 months increasing by 29 species. This effect is different from zero (with a *p*-value<0.05) and robust to the choice of matching algorithm.

**Table 2:** Impact of ComFishIII on number of species caught.

Matching	*N*_*T*_	*N*_*C*_	*ATT*	*s*.*e*.	*t*	95% CI
Nearest neighbor	16	13	29.000	9.220	3.145	9.92 − 46
Kernel	16	16	32.724	8.526	3.838	13.05 − 47.48
Radius (r = 0.05)	16	16	31.058	9.014	3.446	11.62 − 47.60

*N*_*T*_: number of treated (ComFishIII) villages. *N*_*C*_: number of control villages. *ATT*: Average Treatment on the Treated. Bootstrapped standard errors with 1299 repetitions. The Confidence Intervals presented are the 95% Bias-Corrected CI, presenting more conservative estimates of the effect of the program.

### Sensitivity to unobserved confounders

These estimates can be interpreted as causal when the CIA holds. There is no way to directly test this assumption, and instead we present an analysis of the sensitivity of the estimates to the importance of potential unmeasured confounders trough the estimation of Rosenbaum bounds, implemented using the command –rbounds– described in [37]. The results are presented in Table 3.

**Table 3:** Impact of ComFishIII on biodiversity: Rosenbaum bounds.

*e*^*γ*^	sig+	sig-	t-hat+	t-hat-
1	.002	.002	29	29
1.2	.005	.001	26.5	32
1.4	.009	.000	23.5	35
1.6	.016	.000	22.5	36.5
1.8	.024	.000	21	37.5
2	.034	.000	19	38

*e*^*γ*^: log odds of differential assignment due to unobserved factors

sig+: upper bound significance level

sig-: lower bound significance level

t-hat+: upper bound Hodges-Lehmann estimate

t-hat–: lower bound Hodges-Lehmann estimate

Because we mostly worry about positive selection bias (ie, the possibility that the program may have been allocated to areas where it is mostly expected to be beneficial), we mostly care about the possibility that we are over-estimating the *ATT*. For that reason, we focus on the lower values of statistical significance of our estimates (sig+) and the lower bound of *ATT* (t-hat+) under varying importance of potential confounders, as measured by *e*^*γ*^. At the usual cut-off of *e*^*γ*^ = 2 (to be interpreted as “unmeasured confounders importance in explaining treatment status is the double of the importance of measured confounders”), our estimates are still statistically different from 0 at the usual levels of significance (*p*-value = 0.034) and the estimate of *ATT* is still important, with implementation of ComFishIII leading to an average increase in species richness of 19 species.

### Heterogeneity of treatment effects

**Fig 3:**
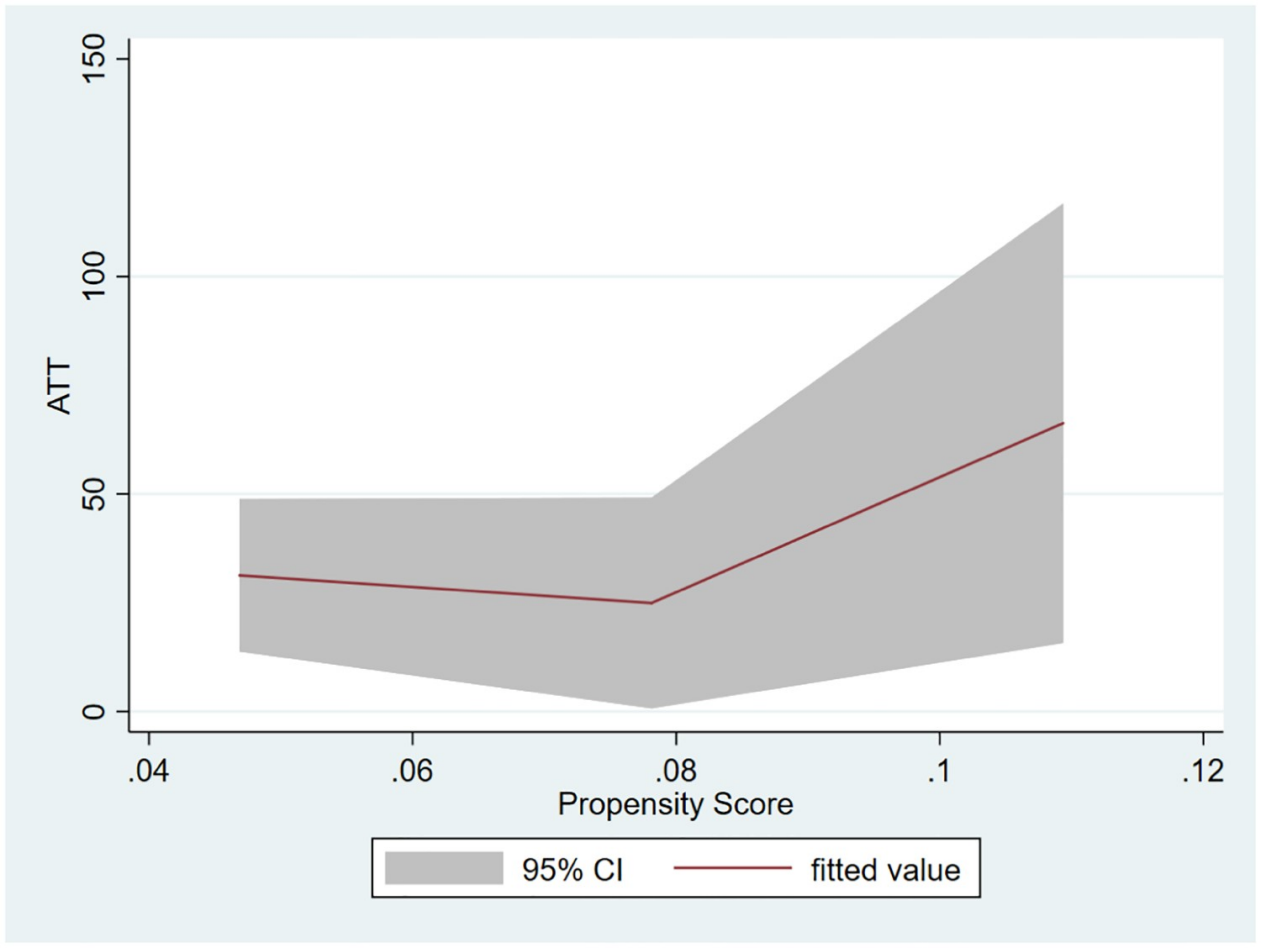
Heterogeneity of impact of the Community Fisheries program (ComFishIII) on biodiversity. Positive values of Average Treatment on the Treated (ATT) indicate an increase in biodiversity.

Finally, in Fig 3 we present the results of the heterogeneity analysis, using the approach outlined in [30], with the *ATT* being re-estimated locally in the neighborhood of each observation (characterized by their propensity score), allowing for an analysis of the relation (trend) between these local estimates and the propensity score. The program is less effective in driving increases in biodiversity in those villages that were least likely to be included in the program (a decision that, recall, mostly reflects the local importance of fishing activities), with large impacts on biodiversity observed in the (few) villages with relatively higher probability of participating in ComFishIII.

## Conclusions and discussion

Understanding whether conservation efforts work, in the sense of what they deliver on their direct environmental objectives, requires a larger effort to incorporate impact evaluation into the design of environmental policies and programs. This article presents evidence that, at least in a context such as the Mekong in Lao PDR, it is possible for freshwater conservation initiatives to achieve improvements in biodiversity.

Our analysis quantifies the causal impact of a fisheries management program, centered on the definition of no-take areas (Fish Conservation Zones) on the number of species caught in the previous 12 months. The effects are large (approximately 29 species), and these estimates are robust to the potential importance of unmeasured confounders, an important concern when using observational data. Our analysis also shows that average impacts are heterogeneous and that initial conditions (as summarized in the probability of participating in the program) matter for the success of this program, suggesting that better targeting of participating villages may further improve its impact.

We conclude with three comments. Firstly, we note that these conclusions are based on a matching design, implemented in a relatively data poor environment. Although we don’t disagree with [15] that there is scope for a larger number of randomized control trials in the evaluation of environmental programs, we also suggest that this approach exemplifies the progress that can be made in their absence [27]. That said, the utility of non-experimental approaches will likely be increased if the evaluation of the impact of a project or program is included from its inception.

Secondly, this article raises the need to clarify the potentially multiple mechanisms through which conservation may impact on biodiversity, a relatively unexplored area of research [38, 39]. Our data suggests that there is substantial heterogeneity in terms of the rules that are implemented in the process of changing fisheries management. Although we have some guidance regarding which design principles may matter (characteristics of the resource, monitoring, punishment rules, …), building on Ostrom’s Socio-Ecological Systems (SES) approach [40], its inherent complexity makes it exceedingly difficult to identify which ones can be understood as essential, particularly in the context of a participatory approach such as the one that underlies ComFishII. This is a clearly important area of future research, with well understood policy implications if one is to “move beyond panaceas” [41, 42], but one that likely requires a larger sample size than what we have available.

Finally, it is perhaps useful to emphasize that, before their wider adoption, FCZ were built on indigenous knowledge of species’ biology and reproduction, the importance of seasonal variation in their habitat and the local importance of spillovers (ie, the increase in fish biomass in areas where fishing is allowed) [11]. Their initial endogenous development likely reflects reliance on local fisheries as a source of protein and micronutrients in the local diet [21, 43, 44]. Such dependency is not unique of Lao PDR, and also characterizes other developing countries [45]. More generally, our analysis provides some encouragement to optimistic views of the relation between poverty reduction and biodiversity conservation [46, 47] that emphasize that the disproportionate reliance of the poor on ecosystem services raises the possibility of synergies between the two objectives [48, 49].

The promise of sustainable development [46, 50] rests on finding ways to identity and achieve such win-win outcomes. That requires to causally establish the links (or lack of) between ecological outcomes, the ecosystem services that potentially flow from them and human outcomes, as they likely drive the continued interest in such conservation initiatives at local level. Even when such analysis is driven by methodological concerns that are similar to those that guide this article, those extra steps bring additional layers of complexity and data requirements—in our case, from establishing the link from freshwater biodiversity to fishing yield [51] and possibly diet variety, to understanding the overall impact of such changes on resource dependent livelihoods [52]—that may also require better integration between social and physical sciences [53].

## Supporting information

S1 Appendix List of fish species & additional results. (PDF)

## References

[1] Dudgeon D, Arthington AH, Gessner MO, Kawabata ZI, Knowler DJ, Lévêque C, et al Freshwater biodiversity: importance, threats, status and conservation challenges. Biological Reviews. 2007;81(2):163–182. 10.1017/S1464793105006950 16336747

[2] Reid AJ, Carlson AK, Creed IF, Eliason EJ, Gell PA, Johnson PTJ, et al Emerging threats and persistent conservation challenges for freshwater biodiversity. Biological Reviews. 2019;94(3):849–873. 10.1111/brv.12480 30467930

[3] Sala OE, Stuart Chapin F, Armesto JJ, Berlow E, Bloomfield J, Dirzo R, et al Global Biodiversity Scenarios for the Year 2100. Science. 2000;287(5459):1770–1774. 10.1126/science.287.5459.1770 10710299

[4] WWF. Living Planet Report 2016: Risk and Resilience in a New Era. WWF International; 2016.

[5] Naiman RJ, Lattterell JJ. Principles for linking fish habitat to fisheries management and conservation. Journal of Fish Biology. 2005;67:166–185. 10.1111/j.0022-1112.2005.00921.x

[6] Fiorella KJ, Bageant ER, Kim M, Sean V, Try V, MacDonell HJ, et al Analyzing drivers of fish biomass and biodiversity within community fish refuges in Cambodia. Ecology and Society. 2019;24(3): article 18. 10.5751/ES-11053-240318

[7] Kiwango Y, Moshi G, Kibasa W, Mnaya B. Papyrus wetlands creation, a solution to improve food security and save Lake Victoria. Wetlands Ecology and Management. 2013;21:147–154. 10.1007/s11273-013-9286-6

[8] Sarkar UK, Pathak AK, Tyagi LK, Srivastava SM, Singh P, Dubey VK. Biodvidersity of freshwater fish of a protected river in India: comparison with unprotected habitat. Revista de Biologia Tropical. 2013; p. 161–172. 10.15517/rbt.v61i1.10942 23894970

[9] van der Ploeg J, Vermeesch L, Rodriguez D, Balbas M, van Weerd M. Establishing freshwater protected areas to protect biodiversity and improve food security in the Philippines In: Westlund L, Charles A, Garcia SM, Sanders J, editors. Marine Protected Areas: interaction with fishery livelihoods and food security. FAO; 2017 p. 31–42.

[10] Baird IG. Integrating Community-Based Fisheries Co-Management and Protected Areas Management in Lao PDR: opportunities for advancemenet and obstacles to implementation; 2000.

[11] Baird IG, Flaherty MS. Mekong River Fish Conservation Zones in Southern Laos: Assessing Effectiveness Using Local Ecological Knowledge. Environmental Management. 2005;36(3):439–454. 10.1007/s00267-005-3093-7 16132441

[12] Ferraro P, Pattanayak S. Money for nothing? A call for empirical evaluation of biodiversity conservation investments. PLOS Biology. 2006;4(4). 10.1371/journal.pbio.0040105 16602825 PMC1435411

[13] Agrawal A, Redford K. Poverty, Development, and Biodiversity Conservation: shooting in the dark?; 2006.

[14] Pullin AS. Why is the evidence base for effectiveness of win-win interventions to benefit humans and biodiversity so poor? Environmental Evidence. 2015;4: article 19. 10.1186/s13750-015-0045-4

[15] Alpizar F, Ferraro P. The environmental effects of poverty programs and the poverty effects of environmental programs: The missing RCTs. World Development. 2020;127: article 104783. 10.1016/j.worlddev.2019.104783

[16] Baylis K, Honey-Rose J, Borner J, Corbera E, Ezzine-de Blas D, Ferraro PJ, et al Mainstreaming impact evaluation in Nature Conservation. Conservation Letters. 2016;9(1):58–64. 10.1111/conl.12180

[17] Ferraro P, Pressey R. Measuring the difference made by conservation initiatives: protected areas and their environmental and social impacts. Philosophical Transactions of the Royal Society B. 2015;370:20140270 10.1098/rstb.2014.0270 PMC4614727 26460123

[18] Ziv G, Baran E, Nam S, Rodríguez-Iturbed I, Levina SA. Trading-off fish biodiversity, food security, and hydropower in the Mekong River Basin. Proceedings of the National Academy of Science. 2012;109:5609–5614. 10.1073/pnas.1201423109 PMC3326487 22393001

[19] Poulse AF, Ouch P, Viravong S, Suntornratana U, Tung NT. Deep pools as dry season habitats in the Mekong River Basin; 2002.

[20] Ounboundisane S, Ainsley S, Patricio H. Evaluation of spillover contribution from Fish Conservation Zones (Freshwater Protected Areas) to village fishing catches in the Nam Kading River, Bolikhamxay Province, Lao PDR; 2014.

[21] Phonvisay S. An Introduction to the Fisheries of Lao PDR. Mekong River Commission; 2013.

[22] DLF, WWF. Fisheries co-management guidelines; 2009.

[23] Cacaud P, Ltadavong P, Funge-Smith S, Kuemlangan B, Meusch E, Mollot R, et al Fisheries and aquaculture in the Lao PDR A legislative review. FAO; 2008.

[24] Angrist JD, Pischke JS. Mostly Harmless Econometrics An Empiricist’s Companion. Princeton University Press; 2009.

[25] Ferraro PJ, Hanauer MM. Advances in Measuring the Environmental and Social Impacts of Environmental Programs. Annual Review of Environment and Resources. 2014;39(1):495–517. 10.1146/annurev-environ-101813-013230

[26] Rosenbaum PR, Rubin DB. The Central Role of the Propensity Score in Observational Studies for Causal Effects. Biometrika. 1983;70(1):41–55. 10.1093/biomet/70.1.41

[27] Agrawal A. Matching and mechanisms in protected area and poverty alleviation research. Proccedings of the National Academy of Science. 2014;111:3909–3910. 10.1073/pnas.1401327111 PMC3964077 24599594

[28] Imbens GW, Rubin DB. Causal Inference for Statistics, Social, and Biomedical Sciences: An Introduction. Cambridge University Press; 2015.

[29] Rosenbaum P. Observational Studies. Springer; 2002.

[30] Xie Y, Jennie E, Jann B. Estimating Heterogeneous Treatment Effects with Observational Data. Sociological Methodology. 2012;41:314–347. 10.1177/0081175012452652 PMC3591476 23482633

[31] Becker SO, Ichino A. Estimation of average treatment effects based on propensity scores. Stata Journal. 2002;2:358–377. 10.1177/1536867X0200200403

[32] Pfaff A, Robalino J. Spillovers from conservation programs. Annual Review of Resource Economics. 2017;9:299–315. 10.1146/annurev-resource-100516-053543

[33] Mascia MB, Fox HE, Glew L, Ahmadia GN, Agrawal A, Barnes M, et al A novel framework for analyzing conservation impacts: evaluation, theory, and marine protected areas. Annals of the New York Academy of Sciences. 2017;1399(1):93–115. 10.1111/nyas.13428 28719737

[34] Gray T, Phommachak A, Vannachomchan K, Guegan F. Using local ecological knowledge to monitor threatened Mekong megafauna in Lao PDR. PLoS ONE. 2017;12(8):e0183247 10.1371/journal.pone.0183247 28820901 PMC5562319

[35] Oliveira AG, Gomes LC, Latini JD, Agostinho AA. Implications of using a variety of fishing strategies and sampling techniques across different biotopes to determine fish species composition and diversity. Natureza & Conservagao. 2014;12(2):112–117. 10.1016/j.ncon.2014.08.004

[36] de Paiva Affonso I, Gomes LC, Agostinho AA, Message HJ, Latini JD, Garcia-Berthou E. Interacting effects of spatial gradients and fishing gears on characterization of fish assemblages in large reservoirs. Reviews in Fish Biology and Fisheries. 2016;26:71–81. 10.1007/s11160-015-9402-1

[37] Gangl M. RBOUNDS: Stata module to perform Rosenbaum sensitivity analysis for average treatment effects on the treated; 2004. Statistical Software Components, Boston College Department of Economics.

[38] Ferraro PJ, Hanauer MM. Through what mechanisms do protected areas affect environmental and social outcomes? Philosophical Transactions Royal Society B. 2015;370:20140267 10.1098/rstb.2014.0267 PMC4614726 26460122

[39] Reimer MN, Haynie AC. Mechanisms matter for evaluating the economic impacts of marine reserves. Journal of Environmental Economics and Management. 2018;88:427–446. 10.1016/j.jeem.2018.01.009

[40] Ostrom E. A General Framework for Analyzing Sustainability of Social-Ecological Systems. Science. 2009;325(5939):419–422. 10.1126/science.1172133 19628857

[41] Ostrom E. A diagnostic approach for going beyond panaceas. Proceedings of the National Academy of Sciences. 2007;104(39):15181–15187. 10.1073/pnas.0702288104 PMC2000497 17881578

[42] Young OR, Webster DG, Cox ME, Raakjær J, Blaxekjær LØ, Einarsson N, et al Moving beyond panaceas in fisheries governance. Proceedings of the National Academy of Sciences. 2018;115(37):9065–9073. 10.1073/pnas.1716545115 PMC6140477 30139919

[43] Jensen J. Traditional fish production: the milk of Southeast Asia. Mekong Fish Catch and Culture. 2001;6(4):1.

[44] Elvevoll EO, James DG. Potential benefits of fish for maternal, foetal and neonatal nutrition: a review of the literature. Food, nutrition and agriculture. 2004;27: online: www.fao.org/docrep/003/X8576M/x8576m05.htm.

[45] Fluet-Chouinard E, Funge-Smith S, McIntyre PB. Global hidden harvest of freshwater fish revealed by household surveys. Proceedings of the National Academy of Sciences. 2018;115(29):7623–7628. 10.1073/pnas.1721097115 PMC6055202 29915069

[46] Barrett CB, Travis AJ, Dasgupta P. On biodiversity conservation and poverty traps. Proceedings of the National Academy of Sciences. 2011;108(34):13907–13912. 10.1073/pnas.1011521108 PMC3161563 21873176

[47] Myers SS, Gaffikin L, Golden CD, Ostfeld RS, H Redford K, H Ricketts T, et al Human health impacts of ecosystem alteration. Proceedings of the National Academy of Sciences. 2013;110(47):18753–18760. 10.1073/pnas.1218656110 PMC3839693 24218556

[48] Benayas JMR, Newton AC, Diaz A, Bullock JM. Enhancement of Biodiversity and Ecosystem Services by Ecological Restoration: A Meta-Analysis. Science. 2009;325(5944):1121–1124. 10.1126/science.1172460 19644076

[49] Kareiva P, Chang A, Marvier M. Development and Conservation Goals in World Bank Projects. Science. 2008;321(5896):1638–1639. 10.1126/science.1162756 18801985

[50] Sachs JD, Baillie JEM, Sutherland WJ, Armsworth PR, Ash N, Beddington J, et al Biodiversity Conservation and the Millennium Development Goals. Science. 2009;325(5947):1502–1503. 10.1126/science.1175035 19762629

[51] Brooks EGE, Holland RA, Darwall WRT, Eigenbrod F. Global evidence of positive impacts of freshwater biodiversity on fishery yields. Global Ecology and Biogeography. 2016;25(5):553–562. 10.1111/geb.12435 27587980 PMC4984834

[52] Robinson EJZ. Resource-Dependent Livelihoods and the Natural Resource Base. Annual Review of Resource Economics. 2016;8(1):281–301. 10.1146/annurev-resource-100815-095521

[53] Ferraro PJ, Sanchirico JN, Smith MD. Causal inference in coupled human and natural systems. Proceedings of the National Academy of Sciences. 2019;116(12):5311–5318. 10.1073/pnas.1805563115 PMC6431173 30126992

