# Supplementary material for: Freshwater fisheries conservation can increase biodiversity
Source: PLoS One. 2020 May 27;15(5):e0233775. doi: 10.1371/journal.pone.0233775 (PMC7252637; doi:10.1371/journal.pone.0233775)
Supplement: S1 Appendix — (PDF) [file pone.0233775.s001.pdf]

Freshwater fisheries conservation can increase biodiversity

Declan Butorac [1], Paulo Santos [2*], Phousavanh Phouvin[3], Francois Guegan [4]

1 Affiliation Environmental Management, Monash University, Clayton, Vic, Australia

2 Affiliation Dept Economics, Monash University, Caulfield, Vic, Australia

3 Affiliation Dept of Fisheries, National University of Laos, Vientiane, Lao PDR

4 Affiliation WWF - Laos, Vientiane, Lao PDR

* paulo.santos@monash.edu

Supporting information

S1 Appendix   List of fish species & Additional results

A Supplementary Information

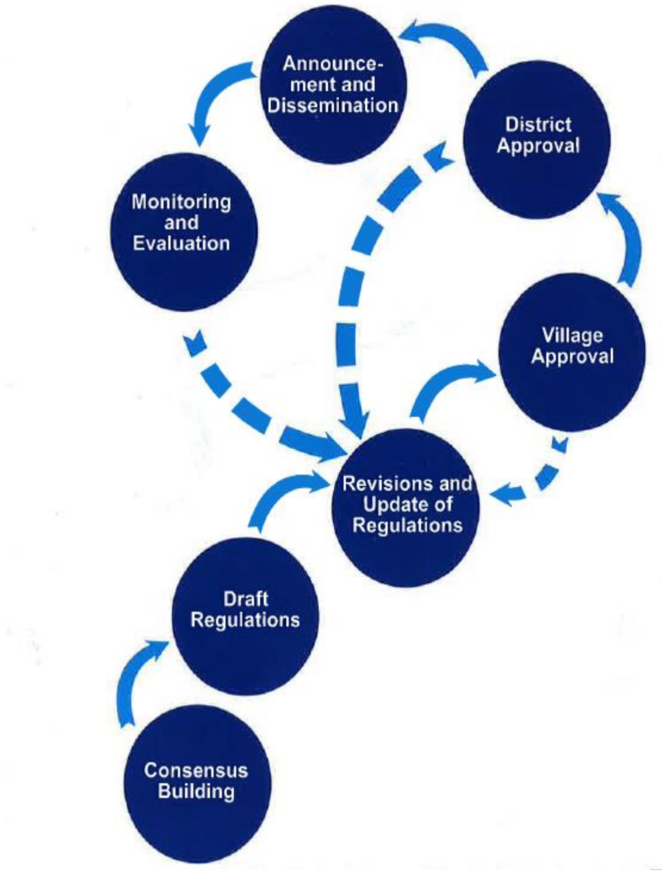

Fig A. Key steps in developing regulations for participatory fisheries management

Table A1. Constructing the comparison group: propensity score estimates

Number of households	0.00173
	(0.00244)
Number of households main income: fisheries	0.0228*
	(0.0137)
Number of households main income: fisheries (squared)	-0.000164
	(0.000224)
Number of households in fishing	0.00918***
	(0.00284)
Number of farm households	-0.0415***
	(0.0158)
Total area of agricultural land	0.000612
	(0.000500)
Number of households with agricultural land	0.0327**
	(0.0149)
Number of households with irrigated land	-0.000739
	(0.00178)
road access, dry season	-0.383*
	(0.211)
road season, both seasons	-0.498**
	(0.199)
distance to primary school	0.0199
	(0.0911)
electricity in village	0.120
	(0.189)
over 70% households have electricity	0.0431
	(0.161)
Number ethnic groups with at least 5% population	-0.0534
	(0.221)
Number of ethnic groups with at least 10% population	0.0553
	(0.288)
Share households - main ethnic group	-0.0111
	(0.0206)
Share households - 2nd main ethnic group	-0.0160
	(0.0186)
District/Province Office	0.636
	(0.455)
resettled village	0.263
	(0.231)
to be resettled village	-0.0550
	(0.293)
Constant	-5.037
	(146.1)
Observations	2,486

Standard errors in parentheses
*** p<0.01, ** p<0.05, * p<0.1

B Supplementary Information: List of species

1. *Notopterus notopterus*
2. *Chitala Blanci*
3. *Chitala Lopis*
4. *Chitala Ornata*
5. *Clupeichthys aesamensis*
6. *Sundasalanx mekongensis*
7. *Achellognathus deignani*
8. *Bangana behri*
9. *Bangana lippus*
10. *Barbonymus gonionotus*
11. *Barbonymus schwanefeldii*
12. *Cirrhinus cirrhosus*
13. *Cirrhinus molitorella*
14. *Cosmochilus harmandi*
15. *Crossocheilus reticulatus*
16. *Cyclocheilichthys apogon*
17. *Cyclocheilichthys armatus*
18. *Cyclocheilichthys enoplos*
19. *Cyclocheilichthys furcatus*
20. *Cyclocheilichthys heteronema*
21. *Cyclocheilichthys repasson*
22. *Cyprinus carpio*
23. *Cyprinus rubrofuscus*
24. *Danio laoensis*
25. *Danio roseus*
26. *Esomus metallicus*
27. *Garra cambodgiensis*
28. *Garra cyclostomata*
29. *Hampala macroplepidota*
30. *Hemibarbus maculatus*
31. *Hysibarbus lagleri*
32. *Hypsibarbus malcolmi*
33. *Hypsibarbus pierrei*
34. *Hypsibarbus vernayi*
35. *Hypsibarbus wetmorei*
36. *Labeo chrysophekadion*
37. *Luciocyprinus striolatus*
38. *Mekongina erythrospila*
39. *Metzia bounthobi*
40. *Mystalacoleucus atridorsalis*
41. *Mystacoleucus cf. marginatus*
42. *Mystacoleucus ectypus*
43. *Mystacoleucus greenwayi*
44. *Mystacoleucus lepturus*
45. *Onychostoma fusiforme*
46. *Onychostoma gerlachi*
47. *Onychostoma meridionale*
48. *Opsarius koratensis*
49. *Opsarius pulchellus*
50. *Osteochilus Lini*
51. *Paralaubuca barroni*
52. *Poropuntius angustus*
53. *Poropuntius carinatus*
54. *Poropuntius laoensis*
55. *Poropuntius normani*
56. *Probarbus jullieni*
57. *Probarbus labeamajor*
58. *Pseudohemiculter dispar*
59. *Pseudorasbora parva*
60. *Puntioplites falcifer*
61. *Puntioplites proctozysro*
62. *Puntioplites waandersi*
63. *Puntius brevis*
64. *Puntius jacobusboehlkei*
65. *Puntius semifasciolatus*
66. *Puntius stoliczkanus*
67. *Raimas guttatus*
68. *Rasbora atridorsalis*
69. *Rhodeus ocellatus*
70. *Scaphiodonichthys acanthopterus*
71. *Scaphognathops stejnegeri*
72. *Scaphognathops theunensis*
73. *Sikukia gudgeri*
74. *Tor tambra*
75. *Tor Tambroides*
76. *Acanthopsoides gracilentus*
77. *Acanthopsoides hapalias*
78. *Botia caudipuncata*
79. *Botia modesta*
80. *Lepidocephalichthys berdmorei*
81. *Annamia normani*
82. *Balitora cf. annamitica*
83. *Hemimyzon papilio*
84. *Hemimyzon pengi*
85. *Nemacheilus longistriatus*
86. *Nemacheilus pallidus*

87. *Nemacheilus platiceps*
88. *Physoschistura meridionalis*
89. *Schistura athos*
90. *Schistura bucculenta*
91. *Schistura melarancia*
92. *Shistura pocali*
93. *Schistura porthos*
94. *Vanmansenia serrilineata*
95. *Hemibargus filamentus*
96. *Hemibagrus aff. nemurus*
97. *Hemibagrus wyckioides*
98. *Belodontichthys truncatus*
99. *Hemisilurus mekongensis*
100. *Kryptopterus bicirrhis*
101. *Micronema apogon*
102. *Micronema bleekeri*
103. *Micronema cheveyi*
104. *Micronema micronemus*
105. *Ompok bimaculatus*
106. *Wallago attu*
107. *Wallago leeri*
108. *Clupisoma sinensis*
109. *Laides longibarbis*
110. *Pangasius macronema*
111. *Pangasius pleurotaenia*
112. *Bagarius bagarius*
113. *Bagarius yarrelli*
114. *Glyptothorax fuscus*
115. *Glyptothorax lampris*
116. *Glyptothorax laosensis*
117. *Glyptothorax macromaculatus*
118. *Akysis inermis*
119. *Clarras batrachus*
120. *Xanentodon canciloides*
121. *Monopterus albus*
122. *Macrognathus siamensis*
123. *Mastacembelus armatus*
124. *Paramassis siamensis*
125. *Pristolepis fasciata*
126. *Oneochromis niloticus*
127. *Oxyeleotris marmorata*
128. *Papuligobius ocellatus*
129. *Rhinogobius nammaensis*
130. *Rhinogobius sp. n. Nam Ou*
131. *Rhinogobius sp.3*
132. *Macropodus opercularis*
133. *Trichogaster trichopterus*
134. *Channa gachua*
135. *Channa striata*
136. *Brachirus harmandi*
137. *Monotrare cambodgiensis*
138. *Monotrate cochinchinensis*
139. *Monotrate turgidus*
